# NFAT5 Contributes to Osmolality-Induced MCP-1 Expression in Mesothelial Cells

**DOI:** 10.1155/2012/513015

**Published:** 2012-02-22

**Authors:** Christoph Küper, Franz-X. Beck, Wolfgang Neuhofer

**Affiliations:** ^1^Department of Physiology, University of Munich, 80336 Munich, Germany; ^2^Department of Nephrology, Medical Clinic and Policlinic IV, University of Munich, Inner City Campus, 80335 Munich, Germany

## Abstract

Increased expression of the C-C chemokine monocyte chemoattractant protein-1 (MCP-1) in mesothelial cells in response to high glucose concentrations and/or high osmolality plays a crucial role in the development of peritoneal fibrosis during continuous ambulatory peritoneal dialysis (CAPD). Recent studies suggest that in kidney cells osmolality-induced MCP-1 upregulation is mediated by the osmosensitive transcription factor, nuclear factor of activated T cells 5 (NFAT5). The present study addressed the question of whether activation of NFAT5 by hyperosmolality, as present in PD fluids, contributes to MCP-1 expression in the mesothelial cell line Met5A. Hyperosmolality, induced by addition of glucose, NaCl, or mannitol to the growth medium, increased NFAT5 activity and stimulated MCP-1 expression in Met5A cells. siRNA-mediated knockdown of NFAT5 attenuated osmolality-induced MCP-1 upregulation substantially. Hyperosmolality also induced activation of nuclear factor-*κ*B (NF-*κ*B). Accordingly, pharmacological inhibition of NF-*κ*B significantly decreased osmolality-induced MCP-1 expression. Taken together, these results indicate that high osmolalities activate the transcription factor NFAT5 in mesothelial cells. NFAT5 in turn upregulates MCP-1, likely in combination with NF-*κ*B, and thus may participate in the development of peritoneal fibrosis during CAPD.

## 1. Introduction

Peritoneal dialysis (PD) is a well-established and effective renal replacement therapy that is employed regularly in patients suffering from end-stage chronic kidney disease. The long-time efficiency of PD is limited by the bio-incompatibility of the currently used PD fluids (PDF) [1]. The latter induces severe pathophysiological changes in the peritoneal membrane, such as fibrosis and angiogenesis, which eventually are responsible for the functional failure of continuous ambulatory peritoneal dialysis (CAPD) [2, 3]. Conventional PDFs are characterized by high concentrations of glucose degradation products (GDPs), an unphysiological low pH, and high osmolalities [4]. In the last two decades significant efforts have been undertaken to improve the biocompatibility of PDFs by minimizing the formation of GDP during heat sterilization [2, 4] and by establishing a more physiological pH [5]. Although the physicochemical properties have been improved, efficient ultrafiltration across the peritoneal membrane requires supraphysiological osmolalities in the range of 380–510 mosm/kg H_2_O, depending on the respective PDF. Accordingly, peritoneal mesothelial cells are exposed to local osmotic stress for several hours during PD.

The assumption that local osmotic stress contributes to the bio-incompatibility of PDFs is supported by the notion that even with the use of novel PDF, with a nearly physiological pH and low content of GDP, the mesothelium produces large amounts of established markers of peritoneal damage [6]. Proinflammatory mediators such as TGF-*β*1 or MCP-1 are synthesized in peritoneal mesothelial cells not only in response to glucose but also in response to osmotic stress [7].

The exposure of mesothelial cells to local osmotic stress during PD suggests an activation of the osmosensitive transcription factor NFAT5 (nuclear factor of activated T cells 5; also known as TonEBP or OREBP) in peritoneal mesothelial cells. NFAT5 was originally identified in collecting duct cells of the renal medulla [8], which are exposed to interstitial osmolalities severalfold higher compared to plasma osmolality (up to 1.200 mosm/kg H_2_O) during antidiuresis. In renal cells NFAT5 regulates the expression of various genes necessary for an efficient urinary concentration, for example, AQP-2 or UT-A, as well as genes required for the adaptation to high osmolalities, for example, aldose reductase or HSP70 [9]. NFAT5-regulated genes contain tonicity enhancer (TonE) elements in their promoter region, to which upon activation NFAT5 binds and stimulates the transcriptional machinery.

Recent studies suggest that under various pathophysiological conditions NFAT5 is activated by local osmotic stress and stimulates the expression of proinflammatory cytokines [10, 11], probably in cooperation with NF-*κ*B [12]. In particular, upregulation of MCP-1 in renal tubular epithelial cells exposed to osmotic stress has been shown to be NFAT5 dependent [12, 13]. The C-C chemokine MCP-1 is a potent chemoattractant for circulating T cells and macrophages/monocytes [14] and plays a key role in the recruitment of these cells to the peritoneal cavity [15]. Besides its chemoattractant activity, MCP-1 stimulates expression of adhesion molecules and proinflammatory cytokines in monocytes [16] and induces calcium flux and the respiratory burst [17]. In fibroblasts, MCP-1 may increase synthesis of collagen and TGF-*β*1 [18], another key factor for the remodelling of peritoneal tissue. Pathophysiological upregulation of MCP-1 expression contributes to fibrotic lesions in the lung [19], the liver [20], and the kidney [21]. Since MCP-1 is also involved in the development of peritoneal damage [22–27], the aim of the present study was to determine whether activation of NFAT5 in response to osmotic stress, as present during PD, contributes to enhanced expression of MCP-1 in peritoneal mesothelial cells.

## 2. Methods

### 2.1. Materials

The NF-*κ*B inhibitor Bay 11-7082 was obtained from Sigma (Deisenhofen, Germany). Anti-NFAT5 antibody weas from Santa Cruz Biotechnology (Santa Cruz, CA, USA); anti-actin antibody was from Sigma; anti-p65 and anti-phospho-p65 (Ser536) and horseradish peroxidase-conjugated anti-rabbit IgG were purchased from Cell Signaling (Beverly, MA, USA); anti-histone H1 was from Millipore (Billerica, MA, USA). Unless otherwise indicated, other reagents were purchased from Biomol (Hamburg, Germany), Biozol (Eching, Germany), Carl Roth (Karlsruhe, Germany), or Sigma.

### 2.2. Cell Culture

Immortalized human mesothelial cells (Met5A, ATCC CRL-9444) were cultured in M199 culture medium supplemented with 4 mM 4-(2-hydroxyethyl)-1-piperazineethanesulfonic acid (HEPES), with 10% fetal bovine serum (Biochrom, Berlin, Germany), 100 units/mL penicillin, and 100 *μ*g/mL streptomycin (Invitrogen, Karlsruhe, Germany) at 37°C in a humidified atmosphere (95% air/5% CO_2_). Cells were grown in 24-well plates to confluency. For experiments, medium osmolality was increased by addition of glucose or, as osmotic controls, NaCl or mannitol.

### 2.3. qRT-PCR Analysis

For determination of MCP-1 and *β*-Actin mRNA expression levels, the total RNA from Met5A cells was prepared by adding TRIFAST Reagent (PEQLAB, Erlangen, Germany). The primers (Metabion, Martinsried, Germany) used in this experiment were MCP-1_fw: 5′-AGT CTC TGC CGC CCT TCT-3′; MCP-1_rev: 5′-GTG ACT GGG GCA TTG ATT G-3′; actin_fw: 5′-CCA ACC GCG AGA AGA TGA-3′; actin_rev: 5′-CCA GAG GCG TAC AGG GAT AG-3′. Experiments were carried out on a Roche LightCycler 480, using the SensiMix SYBR One-Step Kit (Bioline, Luckenwalde, Germany) according to the manufacturer's recommendations. Specificity of PCR product formation was confirmed by monitoring melting point analysis and by agarose gel electrophoresis.

### 2.4. Immunoblot Analysis

Aliquots (5–30 *μ*g protein) were subjected to 10% sodium dodecyl sulfate polyacrylamide gel electrophoresis (SDS-PAGE) and blotted onto nitrocellulose membranes (Amersham Pharmacia Biotech, Buckinghamshire, UK). Nonspecific binding sites were blocked with 5% nonfat dry milk in PBS containing 0.1% Tween-20 (PBS-T) at room temperature for 1 h. Samples were incubated with primary antibodies in PBS-T containing 5% nonfat dry milk over night at 4°C. Subsequently, the blots were washed 3 times with PBS-T for 5 min each, and the membranes then incubated with appropriate secondary antibody at room temperature for 1 h in PBS-T containing 5% nonfat dry milk. After washing with PBS-T 3 times for 5 min each, immunocomplexes were visualized by enhanced chemiluminescence (Pierce, Rockford, IL, USA).

### 2.5. Preparation of Cytosolic and Nuclear Extracts

Subcellular extracts were prepared with the ProteoJET cytoplasmic and nuclear protein extraction kit (Fermentas, St. Leon-Rot, Germany) according to the manufacturer's recommendations, with broad specificity protease inhibitor cocktail (Sigma) added at 1 : 100 (v/v).

### 2.6. MCP-1 Measurement

Concentration of MCP-1 in the cell culture supernatant was determined using a specific ELISA kit (PeproTech, Hamburg, Germany) according to the manufacturer's recommendations.

### 2.7. Reporter Gene Assays

Activation of transcription factors NFAT5 or NF-*κ*B in response to hyperosmolality was assessed using the secreted alkaline phosphatase system (SEAP), with reporter constructs in which the SEAP open reading frame is under control of the respective transacting elements. pNF-*κ*B-SEAP (Clontech, Heidelberg, Germany) contains four copies of the *κ*B response element; pSEAP-TonE contains two TonE sites [28]. For transfection, Met5A cells were grown to ~80% confluency, trypsinated, washed in PBS, and 10^6^ cells were finally resuspended in 200 *μ*L modified HBS electroporation buffer (0.5% HEPES, 1% glucose, 0.5% Ficoll, 5 mM NaCl, 135 mM KCl, 2 mM MgCl_2,_ pH 7.4) together with 10 *μ*g of the respective reporter vector. Electroporation was performed using a Gene Pulser X cell electroporation system (Bio-Rad, Hercules, CA, USA) at 150 V and 950 *μ*F (exponential decay pulse) in a 2 mm cuvette, and the cells were subsequently seeded immediately in 96-well plates. After growing to confluency, the cells were treated as indicated and SEAP activity in the medium determined as described before [29].

### 2.8. NFAT5 Transactivation Assay

NFAT5 transactivation activity was determined using the GAL4 binary assay as initially described by Ferraris et al. [30]. pGAL4-TonEBP-TAD contains the yeast GAL4 DNA-binding domain fused in frame to the transactivation domain (TAD) of NFAT5 (amino acids 548-1531; kindly provided by Dr. J. Ferraris, National Institutes of Health, Bethesda, MD, USA). pFR-SEAP (Agilent Technologies, Santa Clara, CA, USA) contains five tandem repeats of the GAL4 binding site upstream of a minimal promoter and the SEAP gene. Briefly, 10^6^ cells were electroporated with 10 *μ*g pGAL4-TonEBP-TAD and 10 *μ*g pFR-SEAP as described above. After growing to confluency, the cells were treated as indicated and SEAP activity in the medium determined as described before [29].

### 2.9. NFAT5 Knockdown

Accell SMARTpool siRNA construct for knockdown of NFAT5 or Accell nontargeting siRNA (no. 2) were obtained from Thermo Fisher Scientific (Epsom, UK). Knockdown in Met5A cells was performed according to the manufacturer's instructions. The concentration of siRNA constructs was 1 *μ*M in Accell delivery medium, containing 2% FCS. Cells were incubated for 5 days, and knock-down efficiency was determined by qRT-PCR and by western blot analysis.

### 2.10. Statistical Analyses

Data are expressed as means ± SEM. The significance of differences between the means was assessed by Student's *t*-test. *P* < 0.05 was regarded as significant. All experiments were performed at least three times, and representative results are shown.

## 3. Results

### 3.1. Osmolality-Induced Upregulation of MCP-1 Expression in Met5A Cells

**Figure 1:**
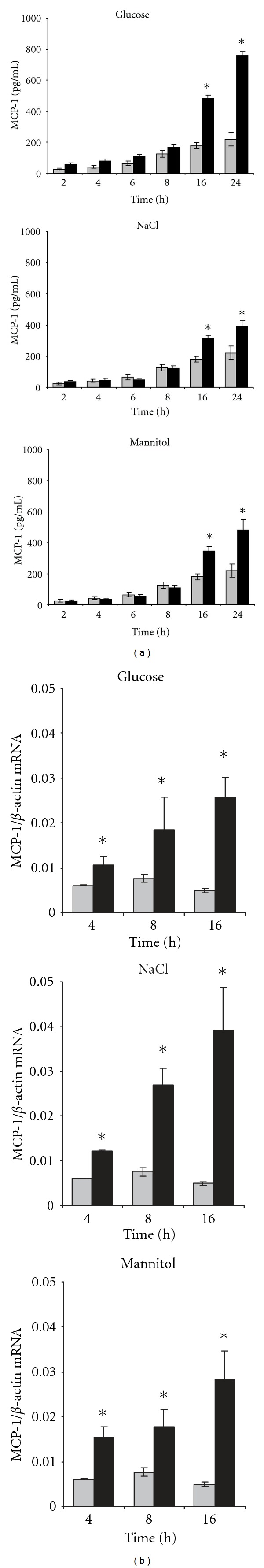
Osmolality-induced MCP-1 expression. (a) For determination of MCP-1 secretion, confluent Met5A cells were incubated in isosmotic medium (gray column; 300 mosm/kg H_2_O) or were exposed to hyperosmotic medium (black column; 400 mosm/kg H_2_O). Medium osmolality was elevated by addition of glucose, NaCl, or mannitol as indicated. At the indicated times, medium samples were collected and the concentration of MCP-1 in the cell culture supernatant determined by ELISA as described in Section 2. Means ± SEM for *n* = 4 per point; *P < 0.05 versus isosmotic control. (b) For determination of MCP-1 transcription, confluent Met5A cells remained in isosmotic medium (gray column; 300 mosm/kg H_2_O) or were exposed to hyperosmotic medium (black column; 400 mosm/kg H_2_O). Medium osmolality was elevated by addition of glucose, NaCl, or mannitol as indicated. At the indicated time points, RNA was extracted from the cells and the abundance of MCP-1 mRNA transcript was determined by qRT-PCR as described in Section 2. Relative MCP-1 mRNA abundance was normalized to that of *β*-actin to correct for differences in RNA input. Data are means ± SEM for *n* = 4 per point; *P < 0.05 versus isosmotic control.

The effect of medium osmolality on MCP-1 secretion of Met5A cells was tested by measurement of MCP-1 concentration in the cell culture supernatants. The cells were incubated in isosmotic (300 mosm/kg H_2_O) or hyperosmotic (400 mosm/kg H_2_O) medium. The medium osmolality was elevated by the addition of glucose. To distinguish between glucose-specific effects and osmolality-induced effects, mannitol or NaCl was used as osmotic controls. Samples of cell culture supernatant were taken at various times between 2 and 24 h. Under isosmotic conditions constitutive MCP-1 secretion could be observed: MCP-1 concentration in the cell culture supernatant rose from 25 ± 8 pg/mL after 2 h to 220 ± 43 pg/mL after 24 h (Figure 1(a)). This constitutive MCP-1 secretion was significantly enhanced under hyperosmotic conditions. The strongest effect was observed when medium osmolality was elevated by addition of glucose: MCP-1 concentration reached 760 ± 26 pg/mL after 24 h. The effects of mannitol (480 ± 67 pg/mL after 24 h) and NaCl (390 ± 36 pg/mL after 24 h) were less pronounced than that of glucose but were still significantly increased compared to the isosmotic control.

An osmolality-induced increase of MCP-1 expression was also observed at the mRNA level (Figure 1(b)). Surprisingly, at all tested times NaCl (rather than glucose) had the strongest effect on MCP-1 mRNA abundance, while on the protein level glucose had the stronger effect (see above), probably indicating that glucose, and not osmolality per se, also stimulates posttranscriptional and/or posttranslational mechanisms, thus further enhancing MCP-1 secretion.

### 3.2. Osmolality-Induced Activation of NFAT5 in Met5A Cells

**Figure 2:**
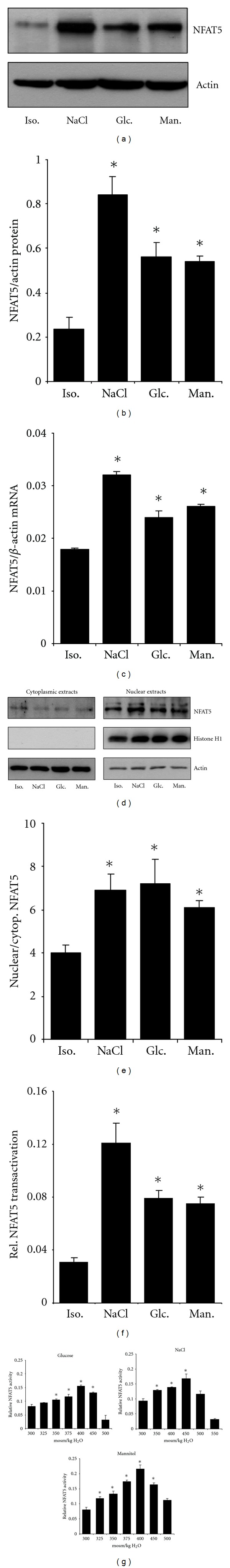
Expression and activation of NFAT5 in Met5A cells. Met5A cells were kept in isosmotic medium (300 mosm/kg H_2_O) or were exposed to hyperosmotic medium (400 mosm/kg H_2_O). Medium osmolality was elevated by addition of glucose, NaCl, or mannitol as indicated. (a) Cells were incubated for 24 h and subsequently processed for immunoblotting as described in Section 2. To demonstrate comparable protein loading, the blots were also probed for actin. A representative blot from 3 independent experiments is shown. (b) Relative NFAT5 protein abundance was quantified by densitometric analysis of immunoblots and normalized to that of actin to correct for differences in protein loading. Means ± SEM for *n* = 3; *P < 0.05 versus isosmotic control. (c) Cells were incubated for 16 h. Thereafter, RNA was extracted and the abundance of MCP-1 mRNA transcript determined by qRT-PCR as described in Section 2. Relative MCP-1 mRNA abundance was normalized to that of *β*-actin to correct for differences in RNA input. Means ± SEM for *n* = 3 per point; *P < 0.05 versus isosmotic control. (d) Cells were incubated for 1 h, and subsequently cytoplasmic and nuclear extracts prepared and processed for immunoblotting as described in Section 2. To demonstrate purity of extracts and comparable protein loading, the blots were also probed for histone H1 and actin. A representative blot from 4 independent experiments is shown. (e) Relative NFAT5 nuclear versus cytoplasmic abundance was quantified by densitometric analysis of immunoblots. Means ± SEM for *n* = 3; *P < 0.05 versus isosmotic control. (f) Activity of the transactivation domain of NFAT5 during osmotic stress. Met5A cells were cotransfected with a vector encoding the fusion protein GAL4dbd-TonEBP-TAD (amino acids 548-1541 of NFAT5 fused to the yeast GAL4 DNA binding domain) together with the reporter vector pFR-SEAP. Cells were kept in isosmotic medium (300 mosm/kg H_2_O) or were exposed to hyperosmotic medium (400 mosm/kg H_2_O). After 48 h, SEAP activity was measured as described in Section 2. Means ± SEM for *n* = 3; *P < 0.05 versus isosmotic control. (g) Met5A cells were transiently transfected with a reporter construct in which the SEAP gene is under control of two TonE sites. Cells were kept in isosmotic (300 mosm/kg H_2_O) medium or were exposed to hyperosmotic medium, with osmolalities between 325 and 550 mosm/kg H_2_O as indicated. After 24 h, SEAP activity was measured as described in Section 2. Means ± SEM for *n* = 4; *P < 0.05 versus isosmotic control.

In kidney cells, hyperosmolality stimulates overall NFAT5 activity by (i) increased NFAT5 expression, (ii) increased NFAT5 translocation into the nucleus, and (iii) activation of the NFAT5 transactivation domain. Accordingly, hyperosmolality elevated NFAT5 expression also in Met5A cells, at both the protein and mRNA levels (Figures 2(a)–2(c)). The most prominent effect was observed when medium osmolality was raised by addition of NaCl; glucose and mannitol also caused a robust increase of NFAT5 abundance. Additionally, hyperosmolality increased translocation of NFAT5 from the cytoplasm into the nucleus (Figures 2(d) and 2(e)) and activity of the NFAT5 transactivation domain (Figure 2(f)).

Activation of NFAT5 in Met5A cells by hyperosmolality was assayed using a TonE-driven reporter vector. Medium osmolalities were elevated to 325–550 mosm/kg H_2_O by addition of glucose, mannitol, or NaCl, and Met5A cells, transfected transiently with the reporter construct, were incubated for 24 h. Raising the medium osmolality increased NFAT5 activity approximately 2-3 fold (Figure 2(g)). For glucose and mannitol, NFAT5 activity reached a maximum at a final osmolality of 400 mosm/kg H_2_O, for NaCl at 450 mosm/kg H_2_O. At even higher osmolalities, NFAT5 activity declined.

### 3.3. Osmolality-Induced MCP-1 Upregulation Is Decreased by NFAT5 Knockdown

**Figure 3:**
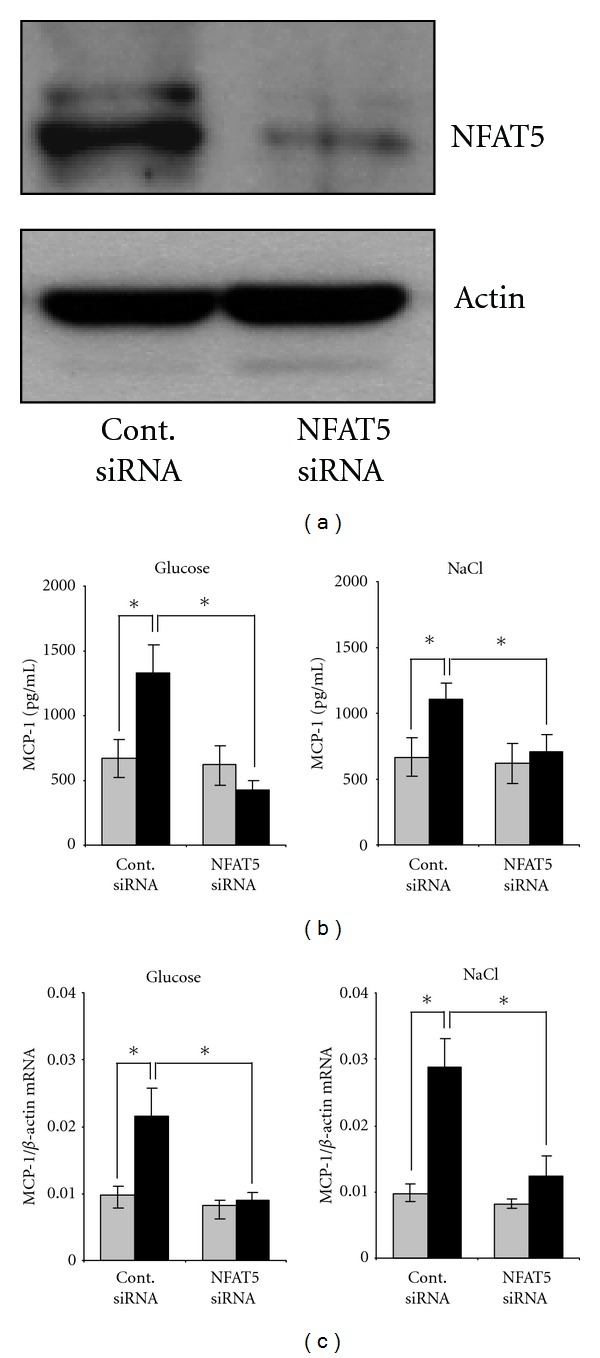
NFAT5-knockdown attenuates osmolality-induced MCP-1 expression. Met5A cells were transfected with siRNA constructs for NFAT5 or with nontargeting siRNA as control as indicated. Cells were kept in isosmotic medium (gray column; 300 mosm/kg H_2_O) or were exposed to hyperosmotic medium (black column; 400 mosm/kg H_2_O). Medium osmolality was elevated by addition of glucose or NaCl as indicated, and cells were incubated for 24 h. (a) To demonstrate efficiency of NFAT5 knockdown, cells were processed for immunoblotting as described in Section 2. To demonstrate comparable protein loading, the blots were also probed for actin. (b) For determination of MCP-1 secretion, medium samples were collected and the concentration of MCP-1 in the cell culture supernatant was determined by ELISA as described in Section 2. Means ± SEM for *n* = 4 per point; *P < 0.05. (c) For determination of MCP-1 transcription, RNA was extracted from the cells and the abundance of MCP-1 mRNA transcript was determined by qRT-PCR as described in Section 2. Relative MCP-1 mRNA abundance was normalized to that of *β*-actin to correct for differences in RNA input. Means ± SEM for *n* = 4 per point; *P < 0.05.

To evaluate the role of NFAT5 in osmolality-induced MCP-1 expression, NFAT5 was knocked down using a siRNA approach. Knock-down efficiency, as tested by immunoblotting (Figure 3(a)), was at ~80% compared with control cells transfected with a scrambled siRNA. As expected, hyperosmolality, induced either by glucose or NaCl addition, significantly increased MCP-1 both in the cell culture supernatant (Figure 3(b)) and at the mRNA level (Figure 3(c)) of control cells. Knockdown of NFAT5 largely attenuated osmolality-induced increase in MCP-1 expression (Figures 3(b) and 3(c)), indicating a central role for NFAT5 in this process.

### 3.4. Role of NF-*κ*B in Osmolality-Induced MCP-1 Expression

**Figure 4:**
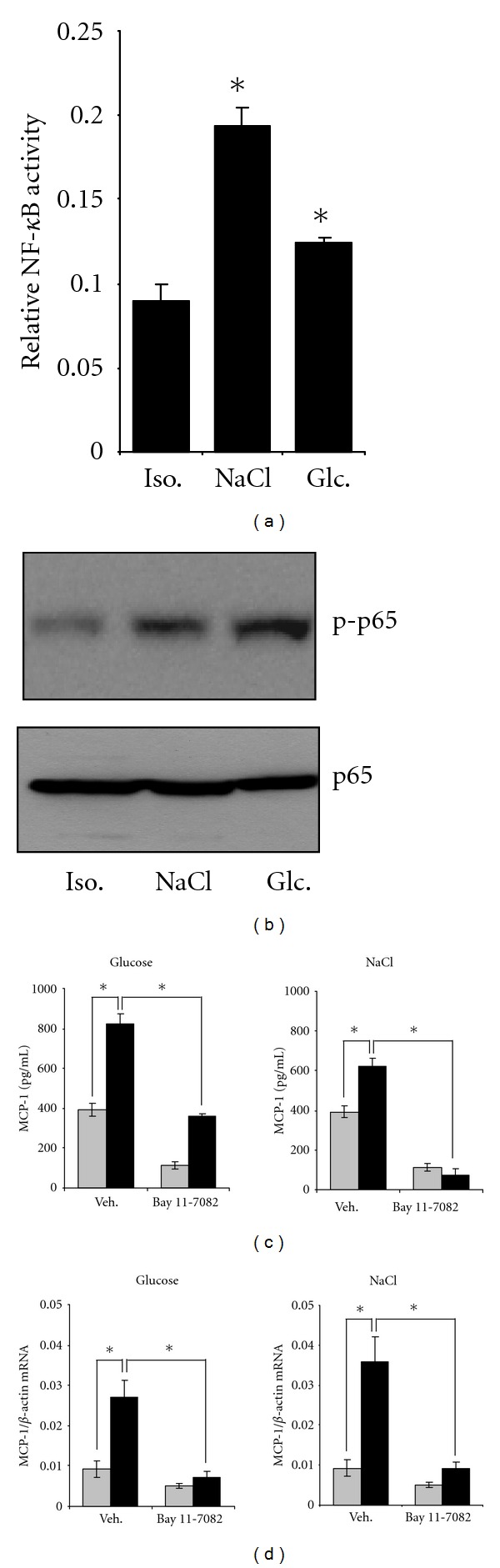
Role of NF-*κ*B in osmolality-induced MCP-1 expression. (a) Activation of NF-*κ*B by osmolality. Met5A cells were transiently transfected with a reporter construct in which the SEAP gene is under control of *κ*B sites. Cells were kept in isosmotic (300 mosm/kg H_2_O) medium or were exposed to hyperosmotic medium (400 mosm/kg H_2_O). Medium osmolality was elevated by addition of glucose or NaCl. After 24 h, SEAP activity was measured as described in Section 2. Means ± SEM for *n* = 4; ^#^ *P* < 0.05 versus isosmotic control. (b) Phosphorylation of the p65 subunit by osmolality. Met5A cells were kept in isosmotic medium (300 mosm/kg H_2_O) or were exposed to hyperosmotic medium (400 mosm/kg H_2_O). Medium osmolality was elevated by addition of glucose or NaCl. After 16 h, cells were processed for immunoblotting as described in Section 2. Abundance of phosphorylated p65 or whole p65 was tested using specific antibodies. A representative blot of three independent experiments is shown. (c) MCP-1 secretion. Met5A cells were preincubated for 1 h with the NF-*κ*B inhibitor Bay 11-7082 (5 *μ*M) or with vehicle DMSO only. Cells were kept in isosmotic (gray column; 300 mosm/kg H_2_O) medium or were exposed to hyperosmotic medium (black column; 400 mosm/kg H_2_O). Medium osmolality was elevated by addition of glucose or NaCl. After 24 h, medium samples were collected and the concentration of MCP-1 in the cell culture supernatant was determined by ELISA as described in Section 2. Means ± SEM for *n* = 4 per point; *P < 0.05. (d) MCP-1 transcription. Met5A cells were preincubated for 1 h with the NF-*κ*B inhibitor Bay 11-7082 (5 *μ*M) or with vehicle DMSO only. Cells were kept in isosmotic (gray column; 300 mosm/kg H_2_O) medium or were exposed to hyperosmotic medium (black column; 400 mosm/kg H_2_O). Medium osmolality was elevated by addition of glucose or NaCl. After 24 h, RNA was extracted from the cells and the abundance of MCP-1 mRNA transcript was determined by qRT-PCR as described in Section 2. Relative MCP-1 mRNA abundance was normalized to that of *β*-actin to correct for differences in RNA input. Means ± SEM for *n* = 4 per point; *P < 0.05.

Another transcription factor probably involved in upregulation of MCP-1 during hyperosmotic stress is NF-*κ*B [27]. In Met5A cells transfected transiently with a *κ*B-driven reporter vector, hyperosmolality, induced by addition of NaCl or glucose, significantly increased reporter activity (Figure 4(a)). Accordingly, phosphorylation of the p65 subunit was enhanced significantly under these conditions (Figure 4(b)), indicating that hyperosmolality activates NF-*κ*B. Next, NF-*κ*B in Met5A cells was inhibited by treatment with the pharmacological inhibitor Bay 11-7082. NF-*κ*B inhibition not only abolished MCP-1 expression under hyperosmotic conditions but also significantly reduced constitutive MCP-1 expression under isosmotic conditions (Figures 4(c) and 4(d)).

## 4. Discussion

Although the biocompatibility of PD solutions has been improved during the recent years by the use of bicarbonate rather than lactate buffers and by limiting excessive GDP formation, hyperosmolality of PDF is required for effective ultrafiltration into the dialysate. The latter is in most cases achieved by the addition of glucose to osmolalities of 380–510 mosm/kg H_2_O. Such high glucose concentrations induce the expression of proinflammatory mediators in mesothelial cells that in turn promote serious long-term complications such as progressive peritoneal fibrosis or even sclerosing peritonitis. Particularly, the expression of TGF-*β*1 and MCP-1 is induced in mesothelial cells in response to high glucose concentrations or high osmolality, respectively [7, 27]. The C-C chemokine MCP-1 recruits monocytes and CD8 T lymphocytes to the peritoneal cavity, where these cells secrete a variety of cytokines and growth factors, which induce or aggravate damage of the peritoneal membrane. In the present study, we provide evidence that the osmosensitive transcription factor NFAT5 contributes to osmolality-induced MCP-1 expression in the mesothelial cell line Met5A. To distinguish between glucose-specific effects and osmolality-induced effects, experiments were carried out not only with glucose but also with NaCl or mannitol as osmotic controls. Generally, activation of NFAT5 and upregulation of MCP-1 were induced by all three agents, indicating that MCP-1 expression is largely induced in response to hyperosmotic stress. However, some differences between NaCl-induced and glucose-induced MCP-1 expression could be observed and are discussed below. siRNA-mediated knockdown of NFAT5 attenuated the osmolality-induced activation of MCP-1 expression, clearly demonstrating the important role for NFAT5 in this context. The regulation of MCP-1 expression under hyperosmotic conditions by NFAT5 has been recently described in rat and human kidney cells [12, 13]. At least one TonE element at position −199 bp to −186 bp upstream from the transcriptional start site has been identified in the MCP-1 regulatory region, to which NFAT5 binds in response to osmotic stress [13]. Activation of NFAT5 under these conditions probably depends on the MAP kinases p38 and ERK1/2 [12, 13].

NFAT5 is reportedly regulated by various mechanisms in response to osmotic stress in kidney cells: by increased NFAT5 expression probably due to stabilization of NFAT5 mRNA [31], by increased activity of the transactivation domain within the c-terminal portion of NFAT5 [30, 32], and by enhanced translocation from the cytoplasm to the nucleus [33, 34]. Accordingly, hyperosmolality induced a significant upregulation of NFAT5 expression, increased nuclear translocation, and an increased activity of the transactivation domain also in Met5A cells.

We demonstrated activation of NFAT5 activity in response to hyperosmolality in Met5A cells using a TonE-driven reporter construct. Generally, the induction of NFAT5 activity by hyperosmolality in Met5A cells is relatively weak compared to kidney cells. With the same TonE-driven reporter construct, we observed an approximately 20-fold induction of NFAT5 activity in kidney cells [28], while in Met5A cells an approximately 2-fold induction occurred. Notably, maximal NFAT5 activation could be observed at about 400–450 mosm/kg H_2_O and declined rapidly at higher osmolalities, while in kidney cells maximal NFAT5 activation is achieved at osmolalities of >500 mosm/kg H_2_O. In kidney cells, the nonreceptor tyrosine kinase focal adhesion kinase (FAK) is a positive regulator of NFAT5 activity under hyperosmotic conditions [35]; in mesothelial cells, high osmolalities, and especially high glucose concentrations, inhibit FAK [36], which may account for the decreasing NFAT5 activity at osmolalities >450 mosm/kg H_2_O. Furthermore, the decreased NFAT5 activity may reflect a general decrease in cellular activity and perhaps cellular damage of mesothelial cells in response to high osmolalities [37]. In contrast, kidney cells have evolved effective mechanisms to maintain cellular activity even during hyperosmolality [38].

Met5A cells showed significant constitutive MCP-1 expression under isosmotic conditions. This basal expression appears to be largely independent of NFAT5, as NFAT5 knockdown had marginal effects on MCP-1 abundance at both the mRNA and protein levels under isosmotic conditions. In contrast, the pharmacological NF-*κ*B inhibitor Bay 11-7082 significantly reduced not only hyperosmotic-induced MCP-1 expression but also basal expression under isosmotic conditions, indicating that basal NF-*κ*B activity under isosmotic conditions is necessary for basal MCP-1 expression. Involvement of NF-*κ*B in regulation of osmolality-induced MCP-1 expression in mesothelial cells has been shown previously [27]. In Met5A cells, hyperosmolality activated a *κ*B-driven reporter vector, especially in response to NaCl but also, to a lesser extent, in response to glucose. Accordingly, hyperosmolality induced phosphorylation of the p65 subunit of NF-*κ*B. A cooperation of NFAT5 with NF-*κ*B during osmolality-induced expression of cytokines has been already demonstrated in kidney cells [12]. In this study, the authors propose a model in which direct interaction of NFAT5 with the p65 subunit of NF-*κ*B increases binding of NF-*κ*B to *κ*B sites around −2470 bp and −2440 bp in the promoter region of MCP-1 and enhances NF-*κ*B transcriptional activity under hypertonic conditions. However, the identification of a TonE site in the MCP-1 promoter region provides evidence that NFAT5 may stimulate MCP-1 expression by two different mechanisms: first, by direct binding to the TonE site around −190 bp and activation of the transcriptional machinery; second, by interaction with NF-*κ*B to enhance DNA binding and transcriptional activity of NF-*κ*B.

Interestingly, there were some differences in MCP-1 upregulation depending on whether medium osmolality was elevated by NaCl or glucose. Addition of NaCl to the medium had a stronger effect on NFAT5 abundance and activity and also on NF-*κ*B activity. Accordingly, MCP-1 mRNA abundance was more robustly induced by addition of NaCl compared to glucose. In contrast, the abundance of MCP-1 protein in cell culture supernatants was significantly more enhanced by glucose than by NaCl, indicating that posttranscriptional and/or posttranslational mechanisms further stimulate MCP-1 accumulation in the presence of glucose. A possible mechanism which may account for this observation could be protein glycosylation. MCP-1 can be modified by O-glycosylation and sialylation [39, 40], and this glycosylation enhances MCP-1 protein stability [41]. Since high glucose concentrations can enhance protein O-glycosylation [42, 43], it is conceivable that enhanced MCP-1 O-glycosylation contributes to the observed increased MCP-1 concentrations in cell culture supernatants under these conditions. However, since O-glycosylation of MCP-1 was not assessed in this study, this possibility remains to be established.

As mentioned above, hyperosmolality activated NFAT5 in mesothelial cells, regardless of whether glucose, NaCl, or mannitol was used as the osmotic agent. This is not surprising since NFAT5 activation by these compounds has been shown before in various cell lines [13, 44, 45]. Several studies indicate that replacement of glucose as osmotic agent by icodextrin or amino acids may improve biocompatibility of PD fluids [46–48]. For future studies, it may be interesting to test the effects of icodextrin- or L-carnitine-induced hyperosmolality on NFAT5 activation and MCP-1 expression in mesothelial cells.

In T cells, NFAT5 has also been identified as a positive regulator for the expression of the proinflammatory cytokines TNF-*α* and lymphotoxin-*β* (LT-*β*) in response to osmotic stress [49]. Since especially TNF-*α* is an important mediator of pathological alterations of the peritoneal membrane during CAPD [50], we also analyzed osmolality-induced expression of TNF-*α* and LT-*β* in Met5A cells. However, we could not detect significant expression of TNF-*α* or LT-*β* either under isosmotic or hyperosmotic conditions (data not shown). This is in accordance with the assumption that, during CAPD, TNF-*α* is synthesized preferentially by monocytes/macrophages [51] rather than mesothelial cells.

The gene encoding the chaperone HSP70 is another NFAT5-regulated gene [52]. Upregulation of HSP70 in response to PDF has been demonstrated in *in vitro* and *in vivo* models of PD [53, 54] and confers increased resistance to PDF toxicity to mesothelial cells [55]. Knockdown of NFAT5 in Met5A cells also decreased HSP70 expression (data not shown), indicating that hyperosmolality-induced NFAT5 activity may also have an important role for cytoprotection of mesothelial cells during CAPD.

## 5. Conclusions

Taken together, the present study indicates that the transcription factor NFAT5 is activated in response to high osmolalities in mesothelial cells and that this activation contributes to increased expression of MCP-1, probably in collaboration with NF-*κ*B.
